# Antimicrobial and Wound Healing Properties of FeO Fabricated Chitosan/PVA Nanocomposite Sponge

**DOI:** 10.3390/antibiotics10050524

**Published:** 2021-05-03

**Authors:** Anbazhagan Sathiyaseelan, Kandasamy Saravanakumar, Arokia Vijay Anand Mariadoss, Myeong-Hyeon Wang

**Affiliations:** Department of Bio-Health Convergence, Kangwon National University, Chuncheon 200-701, Korea; sathiyaseelan.bio@gmail.com (A.S.); saravana732@gmail.com (K.S.); mavijaibt@gmail.com (A.V.A.M.)

**Keywords:** FeO NPs, chitosan, PVA, scaffold, diabetic wound dressing

## Abstract

Diabetic and anemia-associated diabetic wounds increase the considerable morbidity and mortality in people, as reported by clinical studies. However, no anemia-associated diabetic wound dressing materials have been developed until now. Hence, this study aimed to develop a nanocomposite scaffold composed of chitosan (CS), poly (vinyl alcohol) (PVA), and phytogenic iron oxide nanoparticles (FeO NPs), for accelerated anemia-associated diabetic wound healing. The aqueous leaves extract of *Pinus densiflora* (PD) was utilized for the synthesis of iron oxide nanoparticles (FeO NPs). TEM and elemental analysis confirmed smaller size PD-FeO NPs (<50 nm) synthesis with the combination of iron and oxide. In addition, in vitro biological studies displayed the moderate antioxidant, antidiabetic activities, and considerable antibacterial activity of PD-FeO NPs. Further, the different concentrations of PD-FeO NPs (0.01, 0.03, and 0.05%) incorporated CS/PVA nanocomposites sponges were developed by the freeze-drying method. The porous structured morphology and the presence of PD-FeO NPs were observed under FE-SEM. Among nanocomposite sponges, PD-FeO NPs (0.01%) incorporated CS/PVA sponges were further chosen for the in vitro wound-healing assay, based on the porous and water sorption nature. Furthermore, the in vitro wound-healing assay revealed that PD-FeO NPs (0.01%) incorporated CS/PVA has significantly increased the cell proliferation in HEK293 cells. In conclusion, the CS/PVA-PD-FeO NPs (0.01%) sponge would be recommended for diabetic wound dressing after a detailed in vivo evaluation.

## 1. Introduction

Diabetes mellitus (DM) is a class of metabolic disorders caused by the impairment of metabolism in insulin secretion or resistance. In general, diabetes and its associated complications are rising eternally due to the modern lifestyle, including nutrition imbalance, less physical activity, and mental stress [1]. The diabetes condition alters the metabolic function of the kidney, which potentially causes anemia (iron deficiency) [2]. One of the clinical studies reports that iron deficiency can be caused even without anemia [3]. Iron is a major mineral that regulates the key metabolism, such as oxygen supply and energy through iron-containing proteins (myoglobin, hemoglobin, ferritin, and cytochromes), in the human body. Moreover, iron-containing proteins are involved in collagen metabolism through procollagen-proline dioxygenase [4]. Concurrently, iron overload (hemochromatosis), and chronic venous disease (CVD) causes severe complication through specific organ damage in liver, heart, kidney, leg veins, and skin [5]. Diabetes, anemia, and iron deficiency are intently correlated with diabetic wound healing [6,7,8]. Diabetic wound healing is a detrimental impediment in the management of wound healing because of the pathogenic macro and microvascular complications [9,10]. A recent pilot study revealed that 88% of patients’ wounds had not healed completely, due to anemic and/or iron defects [11]. Hence, iron supplementation can improve the wound healing of patients who are affected with chronic ulceration through iron deficiency [12]. Furthermore, it has been reported that iron has moderate α-glucosidase inhibition activity [13].

Wound healing is a complex process which consists of four major phases, including haemostasis, inflammation, proliferation, and remodeling. However, there is a pathophysiological environment and molecular impairment in the diabetic wound healing process, which may delay the healing process that causes acute to chronic condition. Alongside this, nanocomposite material (multicomponent materials which have at least one dimension in the nanoscale) provides multiple preferences to formulate the drug carrier system to increase targeted delivery in the pathological site. In this context, the biodegradable polymer-based (alginates, collagen, chitosan, polyurethane, hyaluronic acid, and pectin) nanocomposites are employed to deliver the drugs to the wound at various conditions and augment the wound healing process [14]. Among the polymers, chitosan (CS) is a unique and cationic linear polysaccharide (deacetylated forms of chitin) used in various biomedical systems [15,16,17]. In addition, several reports have evidenced that chitosan and chitosan-based materials would promote the wound healing process through mucoadhesive and antimicrobial activities [18,19,20]. Furthermore, chitosan-based wound dressing materials have been available commercially, such as QuikClot, HemCon, Celox, and ChitoGauze [21]. However, chitosan scaffold materials are poor in mechanical strength, which leads to limitations in regard to applications. To overcome these difficulties, an earlier study has reported that poly (vinyl alcohol) (PVA) blended CS improved mechanical strength, solubility, and wound healing activity [22,23].

PVA is a hydrophilic synthetic polymer used in several biomedical applications, especially in cartilage and orthopedic treatment because of its chemical resistance, water solubility, and biocompatibility [24]. A recent study reported that CS/PVA hydrogel incorporated cerium oxide nanoparticles improved wound healing efficiency [25]. The iron oxide nanoparticles (FeO NPs) are promising in the treatment of various medical complications, such as anemia, neurodegenerative, cancer, blood contamination, Parkinson’s, and Alzheimer’s disease due to its high surface area, magnetic, photothermal, theragnostic, and biocompatibility properties [26]. Several studies have reported the biosynthesis of FeO NPs with enhanced biological properties (antibacterial, antifungal, antitumor, and immunotherapy) [27,28].

**Scheme 1:**
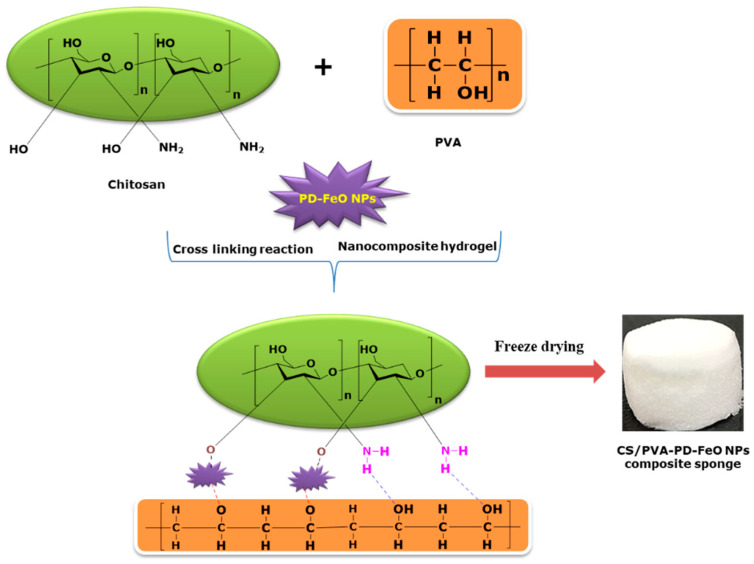
Schematic representation of CS/PVA-PD-FeO NPs composite sponge preparation.

*Pinus densiflora* (PD), generally called Korean red pine tree, is widely distributed in Asian countries, and used as a food ingredient in Korea [29]. PD exhibited significant antioxidant, antitumor, antidiabetic, and antimutagenic properties due to the presence of rich phytocompounds, including proanthocyanidins and catechins [30,31]. Antioxidants protect the cells from hyperglycemia-induced auto-oxidation. They also minimize the micro and macrovascular complications in diabetic conditions. Moreover, there are no studies that have reported on iron oxide nanoparticle incorporated nanocomposite sponges for treatment of anemia-associated diabetic wound healing. Therefore, this work is aimed to the first time to prepare a wound dressing material composed of FeO NPs, CS, and PVA (Scheme 1). Firstly, the FeO NPs were synthesized using PD aqueous leaves extract, and then CS/PVA-PD-FeO NPs nanocomposite sponges were prepared and characterized using various analytical techniques and evaluated for in vitro biocompatibility, cellular glucose uptake, antibacterial antioxidant, and wound healing activity.

## 2. Results and Discussion

The present work reports the green synthesis of multifunctional FeO NPs using the *Pinus densiflora* (PD). We also analyze the PD-FeO NPs incorporated chitosan/PVA nanocomposite wound dressing sponge for anemia-associated diabetic wound healing. The detailed characterization and biological applications of PD-FeO NPs and CS/PVA-PD-FeO NPs sponges are discussed in the below sections.

### 2.1. Characterization

#### 2.1.1. TEM Analysis of PD-FeO NPs

**Figure 1:**
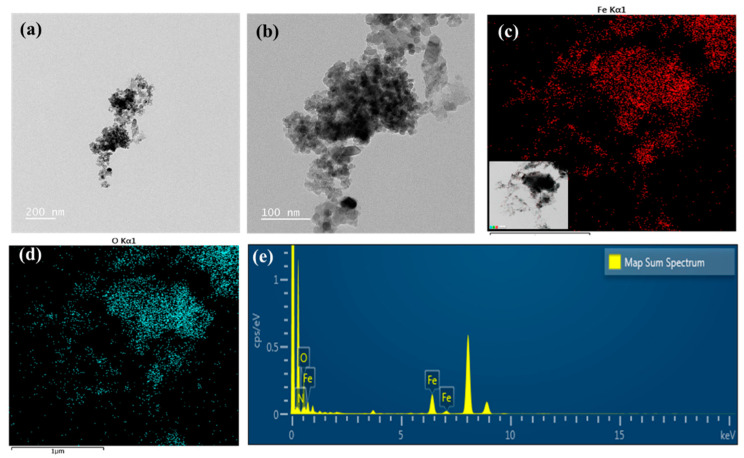
TEM micrograph of PD-FeO NPs. Different magnification of PD-FeO NPs (**a**,**b**), elemental mapping of Fe and O (**c**,**d**), and elemental analysis—(**e**).

TEM micrograph of PD-FeO NPs showed that the uniform size and spherical shape particles surrounded the organic layer, with sizes ranging from 20–50 nm (Figure 1). The aggregation/agglomeration in PD-FeO NPs might be due to the capping of the bioactive molecules, such as polyphenols on the surface of particles, which form the strong bonding between the nanoparticles [32,33]. Similarly, an earlier study has reported that aqueous solution and neutral pH can increase the aggregation in FeO NPs [34,35]. Moreover, the elemental mapping analysis demonstrated the presence of iron and oxygen (Figure 1c–e), which is in accordance with an earlier report [36]. Furthermore, the presence of nitrogen and oxygen in PD-FeO NPs indicates the successful capping of phytomolecules.

#### 2.1.2. Zeta Potential and Particle Size Analysis

**Figure 2:**
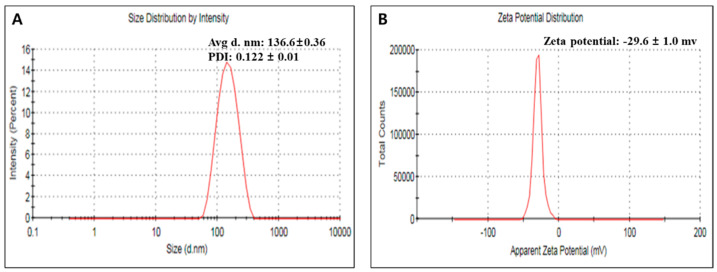
The particle size and zeta potential distribution of PD-FeO NPs. Particle size analysis—(**A**); Zeta potential—(**B**).

Zeta potential and particle size analysis revealed an average size of 136.6 ± 0.36 nm, with a polydispersity index (PDI) of 0.122 ± 0.01 for PD-FeO NPs (Figure 2A). The size differences between TEM and the particle size analyzer depend on the analysis condition. Particle size analysis exhibited the hydrodynamic particle size because the analysis was done in the aqueous medium [37]. Further, PD-FeO NPs exhibited the zeta potential of −29.6 ± 1.0 mV (Figure 2B), which indicated its good stability. The good zeta potential and smaller particles could induce the cell receptor binding and cellular uptake ability [38]. Moreover, the size and zeta potential of the FeO NPs core was influenced by the capping of phytomolecules [39,40].

#### 2.1.3. ATR-FTIR Spectroscopy Analysis

**Figure 3:**
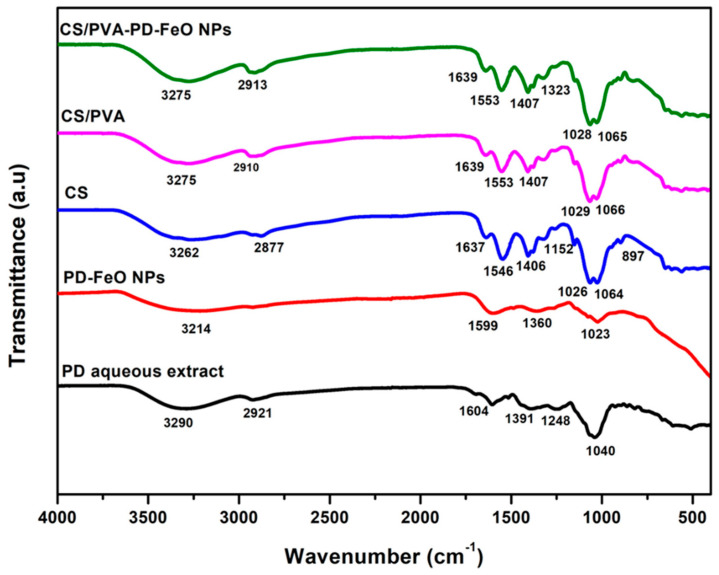
FTIR Spectrum of *Pinus densiflora* (PD) aqueous leaves extract, PD mediated FeO NPs, Chitosan (CS), CS/Poly vinyl alchocol(PVA), CS/PVA-PD-FeO NPs (0.01%).

The functional properties and the interaction of PD aqueous extracts, PD-FeO NPs, CS, CS/PVA, and CS/PVA-PD-FeO NPs (0.01%), were studied through the ATR-FTIR spectroscopy method (Figure 3). PD aqueous extract showed strong broad peaks at 3290 cm^−1^, corresponding to O-H stretching, and medium peaks at 2921 cm^−1^, due to C-H stretching. The medium band at 1604 cm^−1^ is attributable to C=C stretching. The peaks at 1391 cm^−1^ are a result of the O-H bending, and medium peaks at 1248 cm^−1^ are a result of C-N stretching. The strong peaks at 1040 cm^−1^ can be attributed to the C-O stretching of primary alcohol. PD-FeO NPs exhibited strong and broad peaks at 3214 cm^−1^ and 1599 cm^−1^, corresponding to O-H stretching and C=C stretching, respectively. The peaks at 1360 cm^−1^ and 1023 cm^−1^ are responsible for the O-H bending and medium C-N stretching of amine, respectively. The CS exhibited strong and broad peaks at 3262 cm^−1^, which can be attributed to intermolecular O-H stretching, and the medium peaks observed at 2877 cm^−1^ are a result of C-H stretching. Amide I and amide II were observed at 1637 cm^−1^ and 1546 cm^−1^, corresponding to C=O stretching and N-H bending of chitosan [19,41]. Further, the deformation of the CH_3_ band was observed at 1406 cm^−1^ and 1373 cm^−1^ [42]. The PVA exhibited strong broad peaks at 3435 cm^−1^ and 2949 cm^−1^, corresponding to the O-H intermolecular, intramolecular hydrogen bond, and C-H stretching of alkyl groups, respectively. The peaks at 1653 cm^−1^ are a result of C=C stretching, and 1421 cm^−1^ can be attributed to the CH_2_ group [43]. The intermolecular hydrogen bond O-H and C-H stretching at 3275 cm^−1^ and 2910 cm^−1^ was formed respectively in the CS/PVA composites. The shifting of the major peaks was noted when compared to CS alone. Further, the minor shift was observed in the amide I and amide II bands of chitosan at 1639 cm^−1^ and 1553 cm^−1^. The uses of poly(vinyl alcohol) in the scaffold would help to improve the mechanical strength. Moreover, the fabrication of PD-FeO NPs did not exhibit significant changes on CS/PVA composites, because PD-FeO NPs was encapsulated by CS/PVA. Alongside this, the concentration of PD-FeO NPs was lower (0.01%).

#### 2.1.4. XRD Spectrum of CS/PVA-PD-FeO NPs

**Figure 4:**
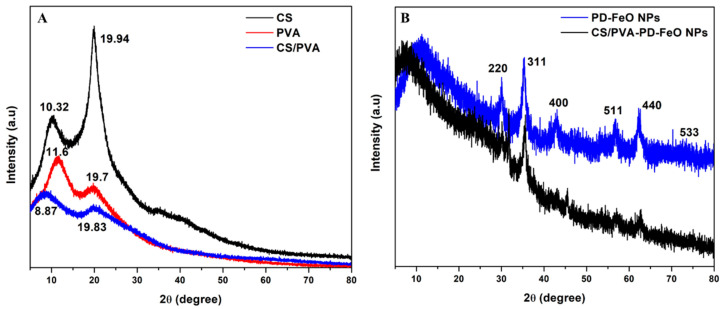
XRD Spectrum. CS, PVA, and CS/PVA—(**A**); PD-FeO NPs, and CS/PVA-PD-FeO NPs—(**B**).

The crystalline properties of CS, PVA, CS/PVA, PD-FeO NPs, and PD-FeO NPs (0.01%) incorporated CS/PVA, were studied through XRD analysis, as shown in Figure 4A,B. The XRD pattern of PD-mediated synthesized FeO NPs was agreed to standard Fe_3_O_4_ (JCPDS: 65-3107). The diffraction peaks of PD-FeO NPs were exhibited at 30.09°, 35.25°, 42.95°, 56.70°, 62.38°, and 74.12°, which corresponds to the 220, 311, 400, 511, 440, and 533 crystal planes of Fe_3_O_4_, which are coherent with earlier reports [44,45]. The CS showed its respective crystalline peaks at 2θ angle 10.32° and 19.94°. Similarly, sigma chitosan was reported as the two crystalline peaks at 10.1° and 19.8° [46]. We also found that sigma chitosan showed major crystalline peaks at 10.0° and 20.1° [47]. Another polymer, PVA, also exhibited the two major crystalline peaks at 2θ angle 11.6° and 19.7°, and the obtained crystal peaks were consistent with earlier reports [48]. The CS blended PVA exhibited the two crystalline peaks at 8.87° and 19.83°, this indicates the conformational transition within the two polymers [49]. Moreover, CS/PVA-PD-FeO NPs (0.01%) composites showed all the crystalline peaks and plane of PD-FeO NPs at 30.09° (220), 35.50° (311), 43.09° (400), 53.55° (422), 57.20° (511), 62.75° (440), and 74.41° (533), while the peak intensity was lowered without any crystalline phase change of PD-FeO NPs (Figure 4B). In addition, earlier reports found that Fe_3_O_4_ nanoparticle crystallinity was not changed by the chitosan coating [50].

#### 2.1.5. SEM

**Figure 5:**
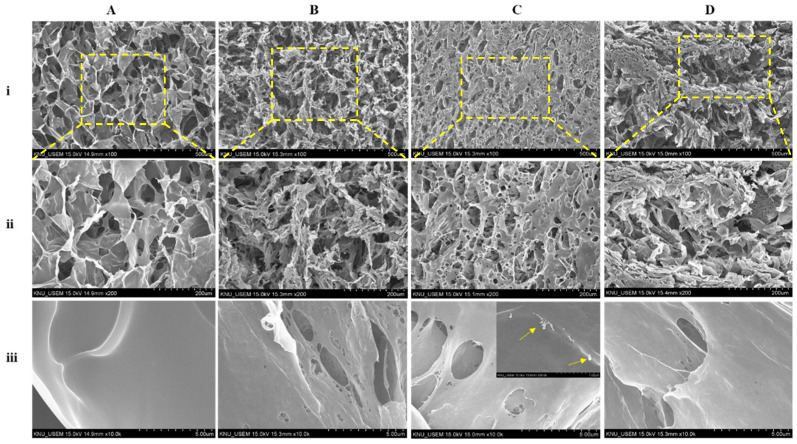
UHR-FE-SEM observation of chitosan—(**A**), Chitosan/PVA sponge—(**B**), PD-FeO NPs (0.01%) incorporated chitosan/PVA sponge, and inner slide shows presence of PD-FeO NPs—(**C**), PD-FeO NPs (0.03%) incorporated chitosan/ PVA sponge—(**D**). Under different magnification such as 500 µm (**i**), 200 µm (**ii**), and 5 µm (**iii**).

The structural morphology of CS, CS/PVA, and PD-FeO NPs (0.01% and 0.03%) incorporated CS/PVA sponges are shown in Figure 5A–D. SEM analysis displayed that CS sponges have a well-interconnected, honey-comb structure, with thin-walled morphology, compared to CS/PVA and CS/PVA-PD-FeO NPs. A dense inter-walled, microporous structure was observed in CS/PVA sponges, which is attributable to the strong hydrogen bonding between the polymer [51]. The 0.01% of PD-FeO NPs incorporated CS/PVA sponges displayed significant dense microporous structured morphology, but this was not observed in CS and CS/PVA sponges. Further, the presence of PD-FeO NPs on the surface was observed under higher magnification (Figure 5(Ciii)). However, when increasing the concentration of PD-FeO NPs to more than 0.01%, the interconnected porous structure of sponges was highly interrupted (Figure 5(Di–iii)). Moreover, the higher concentration of PD-FeO NPs may influence the physical nature of CS/PVA sponges through cross-linking. Accordingly, an earlier study evidenced that the topology and pore size of CS/PVA hydrogel were significantly changed while loading cerium oxide nanoparticles [25].

#### 2.1.6. Porosity

**Figure 6:**
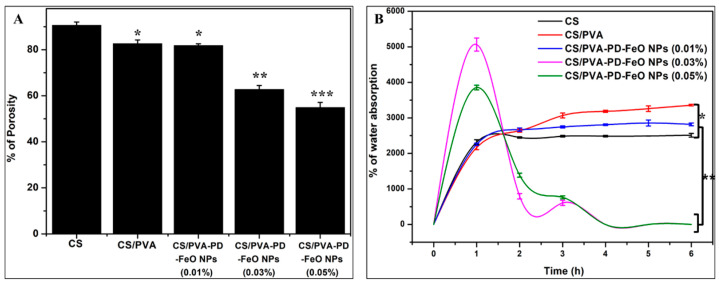
The different concentration of PD-FeO NPs incorporated CS/PVA nanocomposite sponge. Porosity—(**A**); Water absorption—(**B**). * (*p* < 0.05), ** (*p* < 0.01), and *** (*p* < 0.001) significant.

The porosity of CS, CS/PVA, and different concentrations of PD-FeO NPs (0.01, 0.03, and 0.05%) incorporated CS/PVA sponges are shown in Figure 6A. The porosity of various sponges is demonstrated, including CS at 90.57 ± 1.42%, CS/PVA at 82.6 ± 1.61%, CS/PVA FeO NPs (0.01%) at 81.78 ± 0.77%, CS/PVA FeO NPs (0.03%) at 62.72 ± 1.71%, and CS/PVA FeO NPs (0.05%) at 54.88 ± 2.22%. Among them, CS sponges showed the highest porosity, followed by CS/PVA, and 0.01% of PD-FeO NPs incorporated CS/PVA sponges (*p* < 0.001). However, the higher concentration of PD-FeO NPs (0.03 and 0.05%) incorporated sponges exhibited a decreased porosity when compared to CS/PVA-PD-FeO NPs (0.01%) (*p* < 0.05). In our previous study, we found that direct incorporation of silver nanoparticles inhibited a well inter-connected porous structure in fungal chitosan sponges [18]. Highly porous material in wound dressing would help gaseous exchange, excess wound exudate absorption, and maintenance of moist environments [52]. Moreover, moist wound healing supports re-epithelialization and inhibits secondary infections like diabetic condition [53].

#### 2.1.7. Water Absorption

The water absorption behavior of CS, CS/PVA, and the different concentrations of PD-FeO NPs incorporated CS/PVA sponges were determined from 0 to 6 h (Figure 6B). The Chitosan (CS) sponge alone showed a higher water absorption (%) in the 1st h at 2318.28 ± 65.05%, and the water absorption was increased up to 2511 ± 51.71% at 6th h. CS blended PVA sponges demonstrated water absorption at 2163.04 ± 56.56% (1st h), and 3358.91 ± 21.21% (6th h) which is higher than the CS sponge alone, while showing lesser porosity compared to CS. Although the incorporation of PD-FeO NPs (0.03% and 0.05%) in CS/PVA sponges significantly decreased the water absorption after 1 h interval, the 0.01% of PD-FeO NPs incorporation maintained the water absorption capacity constantly. The maximum % of water absorption was found in CS/PVA-PD-FeO NPs (0.01%) at 2855.55 ± 83.80% (6th h). However, 0.03% and 0.05% of PD-FeO NPs incorporated CS/PVA sponges were considerably degraded at the 4th h of the experiment. The water absorption nature highly depends on the properties of the material in composite sponges [54].

### 2.2. Iron Release of CS/PVA-PD-FeO NPs Sponge

**Figure 7:**
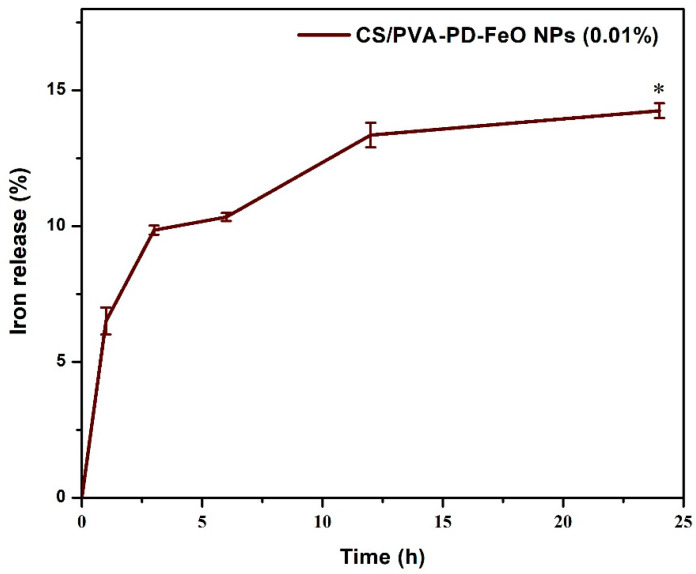
In vitro iron release from CS/PVA-PD-FeO NPs sponges at pH 7.4. * indicates the significant higher release of iron at 24th h comparing to 1st h of interval (*p* < 0.05).

Figure 7 shows the estimated iron release (%) from CS/PVA-PD-FeO NPs (0.01%) at different time intervals (0 to 24 h). The rate of PD-FeO NPs release was significantly increased with the increase in time (*p* < 0.05). The rate of PD-FeO NPs release was 6.5 ± 0.49% at the 1st h and 14.25 ± 0.26% at the 24th h. Overall, the in vitro release pattern indicates the sustained release of iron with a lower percentage until 24 h. It was demonstrated that CS/PVA-PD-FeO NPs negatively impacted the iron release at the pH of 7.4, due to the gelling property of PVA blended chitosan. The lower iron release from nanocomposites is an advantage for the anemia-associated diabetic wound healing process because it avoids iron overload mediated toxicity. However, in vitro iron release may not precisely define the in vivo conditions.

### 2.3. Biological Activities

Biological activities of PD-FeO NPs were determined through antioxidant, antidiabetic, and antibacterial assays. Further, antibacterial, cell viability, and cellular internalization of CS/PVA-PD-FeO NPs composites were evaluated using standard in vitro assays. Moreover, the in vitro wound healing potential of CS/PVA-PD-FeO NPs composites was evaluated in HEK-293 cells. The detailed results and discussions are presented in the following sections.

#### 2.3.1. Antioxidant Properties

The antioxidant potential of PD aqueous extract and PD-FeO NPs was evaluated by DPPH and ABTS^+^ radical scavenging antioxidant assays (Figures S1 and S2). The PD aqueous extract exhibited significant radical scavenging activity in both DPPH and ABTS^+^ radicals, compared to PD-FeO NPs. The maximum tested concentration of 1 mg/mL of PD aqueous extract and PD-FeO NPs showed the percentage of DPPH radical scavenging activity at 75.86 ± 1.3 and 49.11 ± 2.5, respectively. Similarly, the percentage of ABTS radical scavenging activity of PD aqueous extract (1 mg/mL) and PD-FeO NPs (1 mg/mL) was found at 94.86 ± 0.11 and 48.51 ± 0.98, respectively. Ascorbic acid has shown substantial radical scavenging activity against both DPPH and ABTS radicals. The earlier studies have shown that Fe NPs exhibited lower antioxidant activity than *Rhus* and safflower aqueous extract [55] which supports the present results. Moreover, these extracts augment wound healing in the diabetic condition through inhibition of oxidative stress [56].

#### 2.3.2. Antidiabetic Activity

The inhibition of digestive enzymes, such as α-glucosidase and α-amylase, has considerably attenuated the higher blood glucose level in diabetic conditions [57,58], hence the PD aqueous extract and PD-FeO NPs evaluated in vitro α-amylase and α-glucosidase enzyme inhibitory activity (Figures S3 and S4). Like the antioxidant activity, PD aqueous extract (1 mg/mL) exhibited higher digestive enzyme inhibitory activity than the PD-FeO NPs (1 mg/mL). The percentages of α-amylase inhibitory activity of PD aqueous extract and PD-FeO NPs were found at 20.71 ± 4.8 and 18.78 ± 3.14, respectively. In addition, PD aqueous extract and PD-FeO NPs exhibited the percentage of α-glucosidase activity at 79.29 ± 0.48 and 27.01 ± 5.01, respectively. However, PD aqueous extract and PD-FeO NPs significantly inhibit α-glucosidase activity, compared to αamylase. Accordingly, *Sesamum indicum* seeds extract mediated synthesis of iron nanoparticles significantly inhibits the α-amylase comparatively less than acarbose [59].

#### 2.3.3. Antibacterial Activity

Microbial population is common in open wounds, but infective conditions have a detrimental effect on the normal healing process [60]. PD-FeO NPs showed moderate antibacterial activity against tested pathogens, such as *B. cereus*, *S. aureus*, *E. coli*, and *S. enterica*. The minimum inhibitory concentration (MIC) was observed in *S. aureus* (250 µg/mL) while not detected in other tested bacteria (Table 1). Accordingly, earlier studies indicated that FeNPs and FeO NPs have moderately inhibited bacterial growth [61,62,63]. But, CS/PVA incorporated PD-FeO NPs (0.01%) composite sponges showed significant inhibitory activity against all the tested pathogens with MIC (Table 1). Furthermore, the significant zone of inhibition was observed in CS/PVA-PD-FeO NPs (0.01%) composite sponges treated *B. cereus* (22 ± 2 mm), *S. aureus* (21 ± 1 mm), *E. coli* (20 ± 2 mm), and *S. enterica* (22 ± 1.5 mm) (Table 1; Figure S5). However, moderate inhibitory activity was observed in CS/PVA-PD-FeO NPs and compared to the standard antibiotic tetracycline hydrochloride.

**Figure 8:**
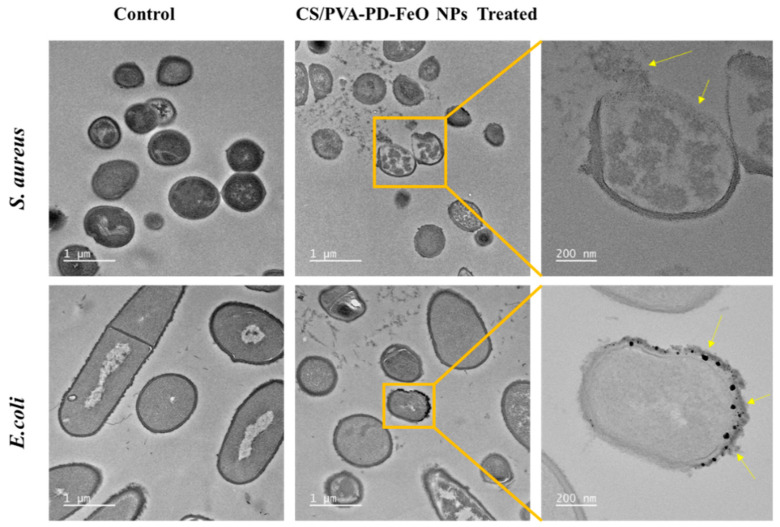
Antibacterial activity of CS/PVA-PD-FeO NPs (0.01%). High-resolution transmission microscopy images of control and treated *S. aureus* and *E. coli*, respectively. The yellow arrows indicate that cytoplasmic leakage and nanoparticle internalization in the bacterial cell.

**Figure 9:**
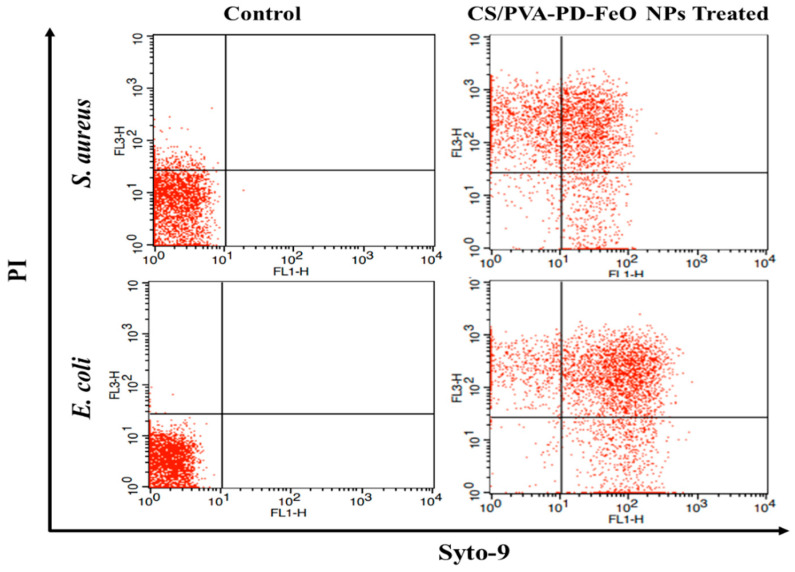
Antibacterial activity of CS/PVA-PD-FeO NPs. Flow cytometry analysis of control and treated *S. aureus* and *E. coli*. Live cells stained with Syto-9 and dead cells stained with Propidium iodide (PI).

The antimicrobial activity of chitosan is highly dependent on the degree of deacetylation, molecular weight, and solubility [64]. The blending of PVA in the nanocomposite turns the chitosan into hydrophilic nature, which exerts antimicrobial properties [65]. To understand the detailed mechanism of the PD-FeO NPs incorporated CS/PVA nanocomposite treated, control bacterial cells was observed in HR-TEM analysis (Figure 8). The untreated *S. aureus* and *E. coli* control bacterial samples maintained the cocci and rod morphology with intact cytoplasm, which seemed to be normal. In contrast, the CS/PVA-PD-FeO NPs treated *S. aureus* cell wall significantly damaged and cytoplasmic leakages were observed, indicated by yellow arrows (Figure 8). Clear morphological changes were observed in the nanocomposite treated *E. coli* cells. The yellow arrows indicate the nanoparticle accumulation and internalization on/onto the cell wall of *E. coli.* The nanocomposites were initiated by the bacterial cell death through structural destabilization and a cytoplasmic leakage mechanism, identified in HR-TEM analysis. Further, the flow cytometry analysis confirmed the considerable bacterial death in nanocomposite treated *S. aureus* (78.6%) and *E. coli* (76.17%), compared to non-treated control cells (Figure 9). Both Syto-9 and PI are referred to as the nuclear stain. Syto-9 could stain both live and dead cells, but PI stained only the cells with damaged membranes [66]. Based on the antibacterial results, CS/PVA-PD-FeO NPs could be the potent antibacterial dressing material that helps to heal wounds by preventing microbial growth.

#### 2.3.4. Cell Viability

**Figure 10:**
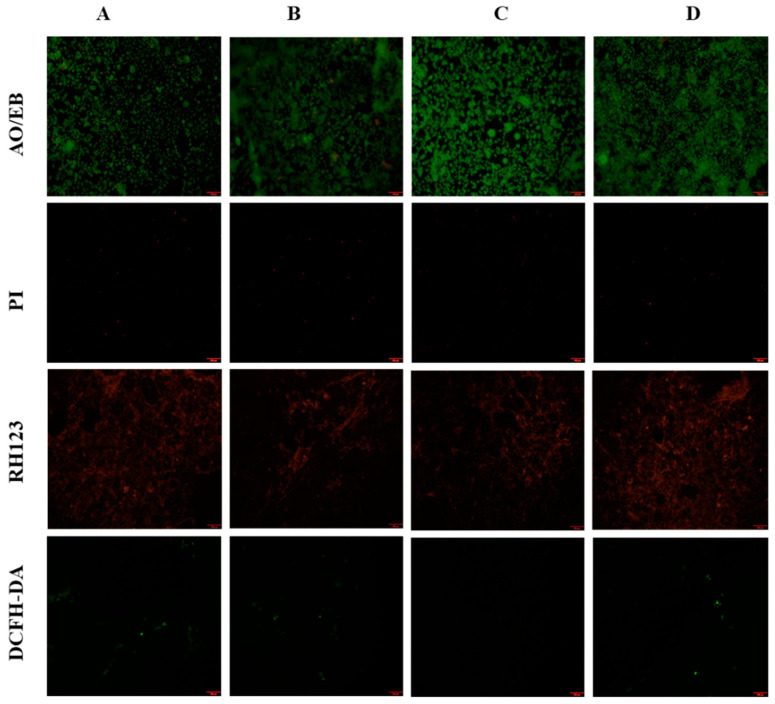
Morphological observation of various fluorescent dye stained HEK 293 cells observed under the fluorescence microscope. Control cells—(**A**); PD-FeO NPs treated cells—(**B**); CS treated cells—(**C**); CS/PVA-PD-FeO NPs treated cells—(**D**).

Biocompatible and less toxic materials can be considered as an ideal wound dressing. Hence, the cell viability of CS, PD-FeO NPs, and CS/PVA-PD-FeO NPs treated HEK-293 cells were evaluated by WST assay (Figure S6) and fluorescent microscopy-based assays (Figure 10). According to the results, the tested samples do not show significant cytotoxicity in HEK-293 cells up to the tested concentration (1 mg/mL). PD-FeO NPs treated cells showed an IC_50_ concentration at 780 ± 20 µg/mL. An earlier study indicates that any tested concentration of iron oxide nanoparticle did not cause toxicity in HUVEC cells up to 24 h incubation, but toxicity was found at 48 h incubation [67]. In the case of CS and CS/PVA-PD-FeO NPs (0.01%), treated samples were not observed to have half of the minimum inhibitory concentration (IC_50_). Several studies reported that chitosan and polyvinyl alcohol showed substantial cell proliferation and a biocompatible nature, which suggests the existence of primary hydroxyl, acetyl, and amine functionalities and its ratio [68,69,70,71,72]. Further, AO/EB fluorescence staining showed a greater number of viable cells in various samples treatment, but in PD-FeO NPs alone, treatment showed fewer apoptotic cells (Figure 10). Similarly, fewer dead cells were observed in PI staining. The CS, CS/PVA, and CS/PVA-PD-FeO NPs treated cells have shown considerable mitochondrial membrane potential (ΔΨm) when compared to the control cells. Then, reactive oxygen species (ROS) production was not observed in the treatment and control cells, which means that the samples did not induce the oxidative stress (Figure 10). Hence, our results conclude that CS/PVA incorporated PD-FeO NPs did not cause any cytotoxicity, and they improve cell viability. Moreover, it can be hypothesized that the fabricated composite decreases the inflammatory cytokines and oxidative enzymes and increases the anti-inflammatory cytokines.

#### 2.3.5. Cellular Glucose Uptake

**Figure 11:**
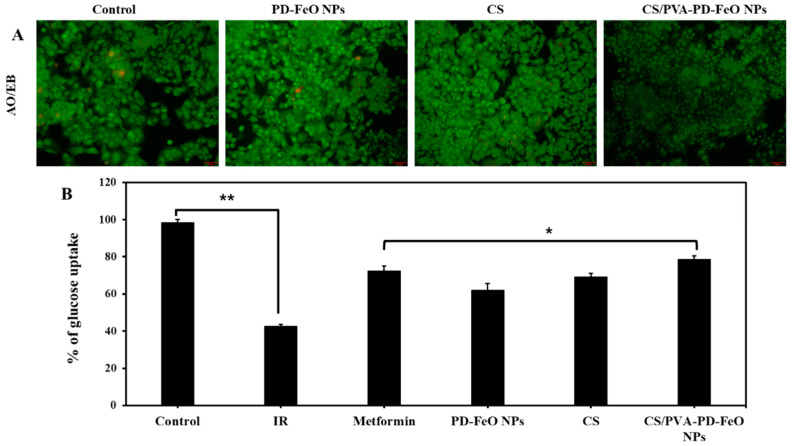
Cell viability and glucose uptake efficiency of nanocomposite treated IR-HepG2 cells. AO/EB staining of various samples treated IR-HepG2 cells—(**A**); glucose uptake efficiency of nanocomposites—(**B**). (** *p* < 0.01), (* *p* < 0.05).

The cell viability and regulation of cellular glucose uptake behavior of PD-FeO NPs, CS, CS/PVA-PD-FeO NPs (0.01%) treated IR-HepG2 cells are shown in Figure 11A,B. The cell viability showed that treatment did not induce any cell toxicity when compared to control observed in AO/EB staining. Further, cellular glucose uptake results showed that CS/PVA-PD-FeO NPs treatment attenuated the insulin resistance, thereby increasing the cellular glucose uptake (78.75 ± 1.7%) in IR-HepG2 cells, when compared to the non-treated IR-HepG2 cells (glucose uptake at 42.52 ± 1.2%). The positive control metformin-treated cells showed a cellular glucose uptake of 72.46 ± 2.4%, which is lesser than CS/PVA-PD-FeO NPs. Similarly, earlier work has reported that superparamagnetic iron oxide nanoparticles (SPION) significantly reduce the blood glucose level in diabetic rats more so than metformin [73]. In addition, SPION was found to regulate the expression of obesity and type-2 diabetic-related genes in human preadipocyte cells [74]. In the present study, the individual treatment of CS and PD-FeO NPs have induced the cellular glucose uptake at 69.07 ± 1.8% and 61.83 ± 3.81%, respectively (Figure 11B). The earlier studies supported the fact that chitosan oligosaccharides and Korean red pine (*Pinus densiflora*) show substantial antidiabetic activity through reduction of blood glucose level [29,75].

#### 2.3.6. Cellular Internalization of PD-FeO NPs

**Figure 12:**
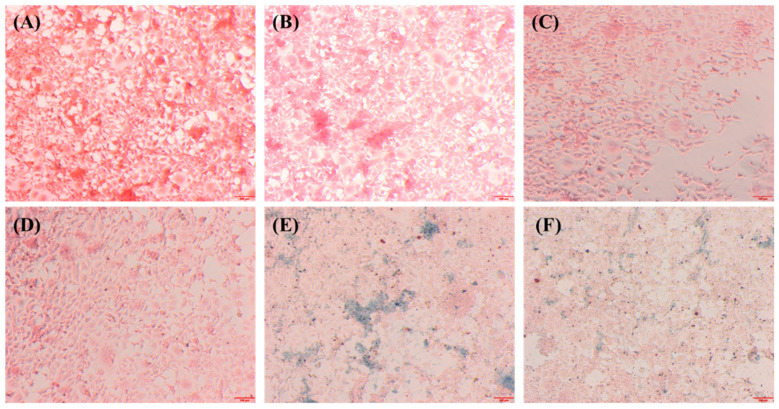
Prussian blue stained PD-FeO NPs cellular internalization in HEK293 cells visualized under the light microscope (10× magnification). The blue staining indicated the presence of PD-FeO NPs. Control—(**A**), chitosan—(**B**), PVA—(**C**), CS/PVA—(**D**), PD-FeO NPs—(**E**), and CS/PVA-PD-FeO NPs—(**F**).

PD-FeO NPs cellular internalization was evaluated in HEK-293 cells by Prussian blue staining counterstained with safranin (Figure 12A–F). For the comparative analysis, HEK293 cells were treated by CS, PVA, CS/PVA, PD-FeO NPs, and CS/PVA-PD-FeO NPs (0.01%). The cellular iron content was only found in PD-FeO NPs and CS/PVA-PD-FeO NPs treated cells (Figure 12E,F), while other treatments and control did not observe any iron content (Figure 12A–D). An early study reports that iron oxide and doxorubicin-loaded chitosan nanoparticles successfully internalized in glioblastoma cells, where iron oxide nanoparticles internalized depending on the concentration observed by Prussian blue staining [37]. The cellular iron oxide nanoparticle uptake significantly enhanced the neurite outgrowth in PC12 cells [76].

#### 2.3.7. In Vitro Wound Healing Assay

**Figure 13:**
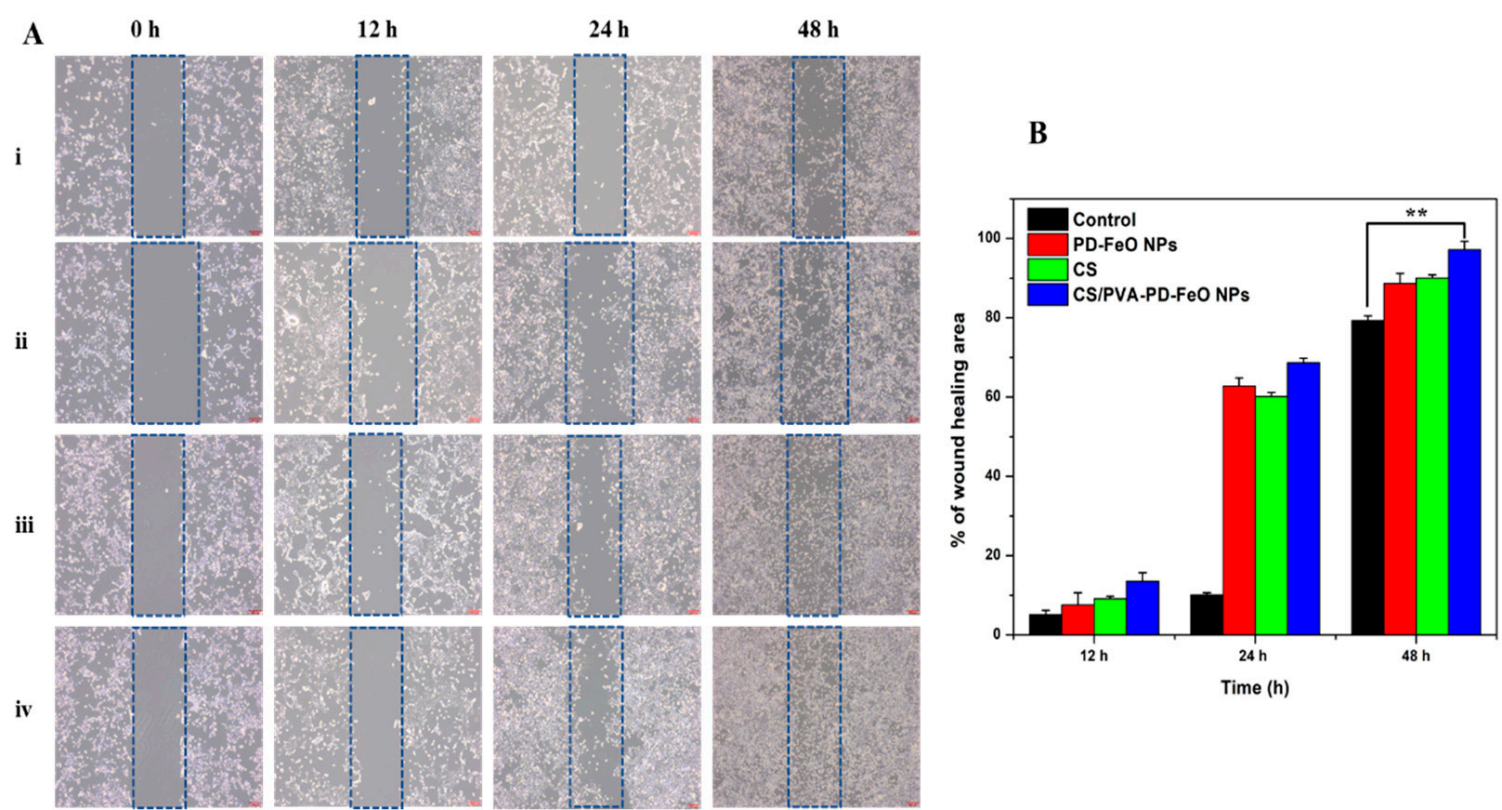
In vitro wound-healing assay. Wound healing efficiency of HEK-123 cells treated with different samples observed under Light microscope—(**A**); Control cells—i; PD-FeO NPs treated cells—ii; CS treated cells—iii; CS/PVA-PD-FeO NPs treated cells—iv. The percentage of wound healing with different treatment—(**B**). ** (*p* < 0.01).

In the in vitro wound-healing assay, we evaluated cell migration and proliferation of mechanically created wounds with and without sample treatment. The different intervals, (0, 12, 24, 48 h) versus sample treatment, (PD-FeO NPs, CS, and CS/PVA-PD-FeO NPs), and control, are shown in Figure 13A. To evaluate the effects of CS/PVA-PD-FeO NPs, the wound area (%) was calculated after 48 h (Figure 13B). The cell migration was seen in 24 h treatment of PD-FeO NPS, CS, CS/PVA-PD-FeO NPs, when compared to control, which had lesser cell migration. Among them, CS/PVA-PD-FeO NPs showed maximum wound area contraction at 48 h (Figure 13(Aiv)), followed by CS, PD-FeO NPs, and control (Figure 13(Ai–iii)). The biocompatible chitosan and PVA provide sustained drug release and metal nanoparticle delivery thereby enhanced cell proliferation and augmented angiogenesis, leading to wound contraction and reduction in cytotoxicity. For example, thrombin conjugated iron oxide nanoparticles enhanced incisional wound healing [77]. Moreover, FeO NPs were found to promote the macrophage autophagy and inflammatory response in cell and animal models [78]. In addition, curcumin-loaded chitosan-based polymeric micelles considerably improve the in vivo wound healing and anti-diabetic effect [79].

## 3. Materials and Methods

### 3.1. Materials

Chitosan (DDA: 75–85%; Mw: 50–190 kDa), ferric chloride hexahydrate (FeCl_3_·6H_2_O), poly(vinyl alcohol) (PVA), sodium hydroxide (NaOH), α-glucosidase (Saccharomyces cerevisiae), α-amylase (porcine pancreas), acarbose, 4-nitrophenyl α-D-glucopyranoside (PNPG), antioxidant (DPPH and ABTS), and starch were procured from Sigma-Aldrich, Republic of Korea. Cell viability assay kit (Cellomax™) was purchased from MediFab, Republic of Korea. L7012 LIVE/DEAD BacLight Bacterial viability kits from ThermoFisher. Acetic acid was procured from the Daejung chemicals & metals co., Ltd., Siheung-si, South Korea. The manufacturer’s information of all the fluorescent staining and culture medium used in the present study is mentioned in our earlier paper. *Pinus densiflora* leaves were collected from Kangwon National University campus, Chuncheon, Republic of Korea.

### 3.2. Synthesis of FeO NPs

Roughly 10 g of fresh leaves of PD were thoroughly washed with distilled water and boiled with 100 mL of distilled water at 80 °C for 20 min. The aqueous PD leaves extract was collected using Whatman filter paper (No. 1) for the synthesis of FeO NPs. Further, the different ratios (1:9, 2:8, 3:7, 4:7, and 5:5) of FeCl_3_ (5 mM) and aqueous PD leaves extract were mixed. Subsequently, NaOH (1 M) was added to the mixture and kept under magnetic stirring for 30 min at 80 °C. The formation of an intense block-colored solution indicated the synthesis of FeO NPs. This was collected by centrifugation at 15,000 rpm, then washed with distilled water, dried at 80 °C, and stored in an airtight container.

### 3.3. Preparation of FeO NPs Fabricated CS/PVA Sponges

The CS, CS/PVA, PVA/CS-PD-FeO NPs composite scaffold was fabricated by the freeze-drying method. For the preparation of the PVA/CS-PD-FeO NPs nanocomposite sponge, 0.5 g of CS was initially dissolved in 100 mL of acetic acid solution (0.5%). Then, an equal volume of water solubilized PVA (5 mg/mL) and CS solution (0.5%) were mixed using magnetic stirring at room temperature for 1 h. The various concentrations of PD-FeO NPs (0.01, 0.03, and 0.05%) were slowly added to the CS/PVA solution under magnetic stirring for 30 min. Finally, well-blended CS, CS/PVA, CS/PVA-PD-FeO NPs (0.01, 0.03, and 0.05%) were freeze-dried. All the developed scaffolds were stored at 4 °C for further characterization and biological assays.

### 3.4. Characterization

The synthesized PD-FeO NPs were initially analyzed using a UV-Visible spectrophotometer (SpectraMax^®^ Plus 384 Microplate Reader from Molecular Devices). The particle size of PD-FeO NPs was observed under a field emission transmission electron microscope (FE-TEM, JEOL-JSM 1200EX Japan). This was followed by particle size distribution, and zeta potential was determined through a particle size Analyzer (Malvern PANalytical Netherland). The presence of phytochemicals, such as total phenol and flavonoids content in PD-FeO NPs, was determined according to the earlier reports [80]. The functional characteristics of PD-FeO NPs, CS, CS/PVA, and CS/PVA-PD-FeO NPs were characterized through FT-IR spectrophotometer (PerkinElmer Paragon 500 USA), and X-ray diffraction analysis (XRD, X’pert-pro MPD-PANalytical Netherland). The surface morphology of CS, CS/PVA, CS/PVA-PD-FeO NPs sponges was observed under an ultra-high-resolution scanning electron microscope (UHR-SEM, Hitachi Japan). The porosity of CS, CS/PVA, CS/PVA-PD-FeO NPs sponges was evaluated by the liquid displacement method, and water sorption capacity was measured according to the earlier report [18].

### 3.5. In Vitro Iron Release

The amount of iron release from the CS/PVA-PD-FeO NPs (0.01%) was determined by ICP-OES (inductively coupled plasm optical emission spectrometer, Agilent 5900) analysis. Firstly, the previously described weight of composite sponges was placed in the 20 mL of phosphate buffer (pH 7.4), in a dissolution bath at 37 °C under shaker (150 rpm). 2 mL of the sample was collected from the dissolution bath at each predetermined time interval (1, 3, 6, 12, and 24 h) and an equal amount buffer solution was replaced to maintain the equal volume. Next, collected samples were diluted to ten-fold for iron quantification, using ICP-OES analysis. The content of iron release was calculated with the known concentration of iron (Correlation coefficient: 0.999) using Agilent Technologies software (Version 7.5.1.11869).

### 3.6. Biological Applications

#### 3.6.1. Antioxidant, Antidiabetic and Antibacterial Assays

The antioxidant (DPPH and ABTS^+^) radical scavenging ability of PD aqueous extract and PD-FeO NPs was determined according to the earlier reports [81]. The antidiabetic potential of PD aqueous extract and PD-FeO NPs was determined in terms of digestive enzyme (α-glucosidase and α-amylase) inhibition, as described earlier [82]. The antibacterial activity of PD-FeO NPs, and CS/PVA-FeO NPs was evaluated against bacterial pathogens, such as *Bacillus cereus* (ATCC 14579), *Staphylococcus aureus* (ATCC 19095), *Escherichia coli* (ATCC 43888), and *Salmonella enterica* (ATCC 14028). The minimum inhibitory concentration of PD-FeO NPs and CS/PVA-PD-FeO NPs were determined by the broth dilution method, according to the earlier report [83]. Furthermore, the antibacterial potential of PD-FeO NPs incorporated CS/PVA composite sponges confirmed in terms of zone of inhibition.

##### High-Resolution Transmission Electron Microscopy (HR-TEM)

The antibacterial effect of CS/PVA-PD-FeO NPs treated each Gram-positive (*S. aureus*) and Gram-negative (*E. coli*) bacteria was determined using HR-TEM (JEOL-JSM 1200EX Japan). To prepare bacteria for HR-TEM analysis, control and treated bacterial cells were initially centrifuged (5000 rpm for 10 min), and the pellet was washed with phosphate buffer (pH 7.4). Further, the pellet was fixed with glutaraldehyde (2.5%) for 4 h, and then samples were dehydrated by 30, 50, 80, and 100% ethanol for 2 h at each step [80]. Finally, air-dried samples were observed under HR-TEM.

##### Flow Cytometry Analysis

The CS/PVA-PD-FeO NPs treated bacterial cells were further evaluated by flow cytometry (FACSCalibur, USA) analysis. For flow cytometry analysis, the bacterial sample and staining (Syto-9 and propidium iodide (PI)) solution were prepared according to the manufacture’s protocol [66].

#### 3.6.2. Cell Viability

The cytotoxicity of PD-FeO NPs, CS, CS/PVA-PD-FeO NPs were evaluated in the human embryonic kidney cell line (HEK-293 cells). The HEK-293 cells were obtained from the Korean cell line bank and cultured in DMEM medium supplemented with FBS (10%) and antibiotics (1%) in a 5% CO_2_ incubator at 37 °C. For the cell viability assay, HEK 293 cells were cultured in 96 well plates for 24 h. After reaching the 70% of confluence, 10 µL of various concentration (3.9, 7.81, 15.62, 31.25, 62.5, 125, 250, 500, 1000 µg/mL) of respective samples were treated to each well and incubated for 24 h in CO_2_ (5%) incubator. After the treatment, 10 µL of WST reagent was added to each well and incubated for 1 h, and then absorbance was measured at 450 nm. The percentage of cell viability was calculated using the following Equation (1).

% of cell viability = (Treated cells absorbance @ 450 nm)/(Control cells absorbance @ 450 nm) × 100(1)

Further, cytotoxicity of PD-FeO NPs, CS, CS/PVA-PD-FeO NPs treated HEK 293 cells was evaluated under a fluorescence microscope using various fluorescence staining (AO/EB, PI, RH123, and DCFH-DA).

#### 3.6.3. In Vitro Glucose Uptake

Cellular glucose uptake efficiency of PD-FeO NPs, CS, and CS/PVA-PD-FeO NPs was determined in insulin-resistant hepatocellular carcinoma (IR-HepG2) cells according to the earlier report [84,85].

#### 3.6.4. In Vitro Wound Healing Activity

The effect of PD-FeO NPs (0.01%), CS (0.5%), and CS/PVA-PD-FeO NPs (0.01%) treatments on wound healing activity was evaluated by in vitro scratch assay [86]. HEK-293 cells were cultured in six-well plates in a CO_2_ incubator at 37 °C, and we allowed cells to reach the proper monolayer confluence. Then, the artificial wound was created by scarping the cell monolayer in a straight line, using a pipette tip (200 µL), and cell debris was washed with PBS. The wounded cell monolayer was treated with each sample incorporated DMEM medium, while the control cells received the DMEM medium alone and were incubated in a CO_2_ incubator. This was followed by the wound healing being observed under a light microscope at each predetermined time interval (0 h, 6 h, 12 h, 24 h, and 48 h). The wound area was measured by ImageJ software. Further, the percentage of wound healing was calculated by the following Equation (2).

% of wound healing = (wound area on the initial day − wound area on the final day)/(wound area on the initial day) × 100(2)

#### 3.6.5. PD-FeO NPs Cellular Internalization

The cellular internalization of FeO NPs was observed by Prussian blue staining assay according to an earlier report [87], with some modifications. In brief, CS, CS/PVA, PD-FeO NPs (0.01%) and CS/PVA-PD-FeO NPs (0.01%) treated HEK-293 cells were incubated for 24 h at a 5% CO_2_ incubator. After incubation, cells were washed with PBS twice, to remove the excess amount of FeO NPs. Then cells were stained with a 1:1 ratio of K_4_Fe(CN)_6_ (10%) and HCl (20%) for 20 min in the incubator. Further cells were counterstained with safranin and washed with 0.02% of acetic acid three times and then observed under a light microscope at 10× magnification.

### 3.7. Statistical Analysis

All of the experimental data were presented as mean ± standard deviation. The one-way analysis of variance (ANOVA) was used for the statistical analysis. The *p*-value < 0.05 was considered statistically significant.

## 4. Conclusions

The results reveal that PD aqueous extracts have a higher amount of total phenol and flavonoid resulting in significant antioxidant and antidiabetic activity. The PD aqueous extract-mediated iron oxide NPs exhibited good biological activity. Further, the bioactive potential of iron oxide NPs (0.01%) loaded CS/PVA nanocomposite sponge displayed highly porous properties, and potential water sorption properties. The sustained and lower iron release (%) was observed from nanocomposite sponges. CS/PVA-PD-FeO NPs (0.01%) exhibited a substantial antibacterial activity against tested bacterial pathogens. Further, CS/PVA-PD-FeO NPs (0.01%) augmenting cell proliferation in HEK-293 cells observed in in vitro wound healing scratch assay. The plant-mediated synthesized iron oxide nanoparticles, chitosan, and poly (vinyl alcohol) consisted of nanocomposite sponges (CS/PVA-PD-FeO NPs) which supported gaseous exchange, wound exudated absorption, and microbial inhibition in the diabetic wound. Hence, we conclude that the existence of antioxidant, antidiabetic, and antibacterial properties of CS/PVA-PD-FeO NPs would aid diabetic wound healing. However, further in vivo studies are required to evaluate the molecular mechanism of diabetic wound healing of developed sponges.

## Figures and Tables

**Table 1:** Minimum inhibitory activity of PD-FeO NPs and PD-FeO NPs incorporated CS/PVA nanocomposites. Values represent the mean ± SD of three replicates. Values share the common superscript alphabets not significant between the samples (*p* < 0.05). TCH-Tetracycline hydrochloride; ND-Not detectable.

Samples	*B. Cereus*	*S. Aureus*	*E. Coli*	*S. Enterica*
	Minimum Inhibitory Concentration (µg/mL)			
PD-FeO NPs	ND	250 ^d^	ND	ND
CS	62.5 ^c^	62.5 ^c^	125 ^c^	62.5 ^c^
CS/PVA-PD-FeO NPs	31.2 ^b^	31.2 ^b^	62.5 ^b^	62.5 ^b^
TCH	7.8 ^a^	7.8 ^a^	15.6 ^a^	15.6 ^a^
	Zone of inhibition (mm)			
CS/PVA-PD-FeO NPs	22 ± 2	21 ± 1	20 ± 2	22 ± 1.5

## Data Availability

The data presented in this study are available on request.

## Supplementary Materials

The following are available online at https://www.mdpi.com/article/10.3390/antibiotics10050524/s1, Figure S1: DPPH radical scavenging activity, Figure S2: ABTS radical scavenging activity, Figure S3: Alpha-glucosidase enzyme inhibitory activity, Figure S4: Alpha-amylase enzyme inhibitory activity, Figure S5: Cell viability.

## References

[1] Sami W., Ansari T., Butt N.S., Hamid M.R.A. (2017). Effect of diet on type 2 diabetes mellitus: A review. Int. J. Health Sci..

[2] Mehdi U., Toto R.D. (2009). Anemia, diabetes, and chronic kidney disease. Diabetes Care.

[3] Soppi E.T. (2018). Iron deficiency without anemia—A clinical challenge. Clin. Case Rep..

[4] Wright J.A., Richards T., Srai S.K.S. (2014). The role of iron in the skin and cutaneous wound healing. Front. Pharmacol..

[5] Zamboni P. (2006). The Big Idea: Iron-dependent inflammation in venous disease and proposed parallels in multiple sclerosis. J. R. Soc. Med..

[6] Ferris A.E., Harding K.G. (2019). An overview of the relationship between anaemia, iron, and venous leg ulcers. Int. Wound J..

[7] Shareef A.M., Ahmedani M.Y., Waris N. (2019). Strong association of anemia in people with diabetic foot ulcers (DFUs): Study from a specialist foot care center. Pak. J. Med. Sci..

[8] Gezawa I.D., Ugwu E.T., Ezeani I., Adeleye O., Okpe I., Enamino M. (2019). Anemia in patients with diabetic foot ulcer and its impact on disease outcome among Nigerians: Results from the MEDFUN study. PLoS ONE.

[9] Okonkwo U.A., Dipietro L.A. (2017). Diabetes and wound angiogenesis. Int. J. Mol. Sci..

[10] Chawla A., Chawla R., Jaggi S. (2016). Microvasular and macrovascular complications in diabetes mellitus: Distinct or continuum?. Indian J. Endocrinol. Metab..

[11] Ferris A.E., Harding K.G. (2020). Does localized iron loss in venous disease lead to systemic iron deficiency? A descriptive pilot study. Wound Repair Regen..

[12] Cappellini M.D., Comin-Colet J., de Francisco A., Dignass A., Doehner W., Lam C.S., Macdougall I.C., Rogler G., Camaschella C., Kadir R. (2017). Iron deficiency across chronic inflammatory conditions: International expert opinion on definition, diagnosis, and management. Am. J. Hematol..

[13] Wang Y., Ma L., Li Z., Du Z., Liu Z., Qin J., Wang X., Huang Z., Gu L., Chen A.S.C. (2004). Synergetic inhibition of metal ions and genistein on α-glucosidase. FEBS Lett..

[14] Boateng J.S., Matthews K.H., Stevens H.N.E., Eccleston G.M. (2008). Wound Healing Dressings and Drug Delivery Systems: A Review. J. Pharm. Sci..

[15] Aranaz I., Mengibar M., Harris R., Panos I., Miralles B., Acosta N., Galed G., Heras A., Aranaz I., Mengíbar M. (2009). Functional Characterization of Chitin and Chitosan. Curr. Chem. Biol..

[16] Ong T.H., Chitra E., Ramamurthy S., Ling C.C.S., Ambu S.P., Davamani F. (2019). Cationic chitosan-propolis nanoparticles alter the zeta potential of *S. Epidermidis*, inhibit biofilm formation by modulating gene expression and exhibit synergism with antibiotics. PLoS ONE.

[17] Rinaudo M. (2006). Chitin and chitosan: Properties and applications. Prog. Polym. Sci..

[18] Sathiyaseelan A., Shajahan A., Kalaichelvan P.T., Kaviyarasan V. (2017). Fungal chitosan based nanocomposites sponges—An alternative medicine for wound dressing. Int. J. Biol. Macromol..

[19] Sathiyaseelan A., Kalaichelvan P.T. (2018). Application of tetracycline hydrochloride loaded-fungal chitosan and *Aloe vera* extract based composite sponges for wound dressing. J. Adv. Res..

[20] Qu J., Zhao X., Liang Y., Zhang T., Ma P.X., Guo B. (2018). Antibacterial adhesive injectable hydrogels with rapid self-healing, extensibility and compressibility as wound dressing for joints skin wound healing. Biomaterials.

[21] Khan M.A., Mujahid M. (2019). A review on recent advances in chitosan based composite for hemostatic dressings. Int. J. Biol. Macromol..

[22] Chen C., Liu L., Huang T., Wang Q., Fang Y. (2013). Bubble template fabrication of chitosan/poly(vinyl alcohol) sponges for wound dressing applications. Int. J. Biol. Macromol..

[23] Rafique A., Mahmood Zia K., Zuber M., Tabasum S., Rehman S. (2016). Chitosan functionalized poly(vinyl alcohol) for prospects biomedical and industrial applications: A review. Int. J. Biol. Macromol..

[24] Baker M.I., Walsh S.P., Schwartz Z., Boyan B.D. (2012). A review of polyvinyl alcohol and its uses in cartilage and orthopedic applications. J. Biomed. Mater. Res. Part B Appl. Biomater..

[25] Kalantari K., Mostafavi E., Saleh B., Soltantabar P., Webster T.J. (2020). Chitosan/PVA hydrogels incorporated with green synthesized cerium oxide nanoparticles for wound healing applications. Eur. Polym. J..

[26] Bruschi M.L., de Toledo L.d.A.S. (2019). Pharmaceutical Applications of Iron-Oxide Magnetic Nanoparticles. Magnetochemistry.

[27] Alphandéry E. (2020). Iron oxide nanoparticles for therapeutic applications. Drug Discov. Today.

[28] Aisida S.O., Akpa P.A., Ahmad I., Zhao T.K., Maaza M., Ezema F.I. (2020). Bio-inspired encapsulation and functionalization of iron oxide nanoparticles for biomedical applications. Eur. Polym. J..

[29] Min H.J., Kim E.J., Shinn S.W., Bae Y.S. (2019). Antidiabetic activities of Korean red pine (*Pinus densiflora*) inner bark extracts. J. Korean Wood Sci. Technol..

[30] Kwak C.S., Moon S.C., Lee M.S. (2006). Antioxidant, antimutagenic, and antitumor effects of pine needles (*Pinus densiflora*). Nutr. Cancer.

[31] Roh S.-G., Choi W.-C. (2011). Antidiabetic Synergistic Effects of Medicinal Plant Extract Mixtures on db/db Mice. J. Life Sci..

[32] Bibi I., Nazar N., Ata S., Sultan M., Ali A., Abbas A., Jilani K., Kamal S., Sarim F.M., Khan M.I. (2019). Green synthesis of iron oxide nanoparticles using pomegranate seeds extract and photocatalytic activity evaluation for the degradation of textile dye. J. Mater. Res. Technol..

[33] Vasantharaj S., Sathiyavimal S., Senthilkumar P., LewisOscar F., Pugazhendhi A. (2019). Biosynthesis of iron oxide nanoparticles using leaf extract of *Ruellia tuberosa*: Antimicrobial properties and their applications in photocatalytic degradation. J. Photochem. Photobiol. B Biol..

[34] Kannan D., Yadav N., Ahmad S., Namdev P., Bhattacharjee S., Lochab B., Singh S. (2019). Pre-clinical study of iron oxide nanoparticles fortified artesunate for efficient targeting of malarial parasite. EBioMedicine.

[35] Herlekar M., Barve S., Kumar R. (2014). Plant-Mediated Green Synthesis of Iron Nanoparticles. J. Nanopart..

[36] Vinayagam R., Zhou C., Pai S., Varadavenkatesan T., Narasimhan M.K., Narayanasamy S., Selvaraj R. (2021). Structural characterization of green synthesized magnetic mesoporous Fe_3_O_4_NPs@ME. Mater. Chem. Phys..

[37] Gholami L., Tafaghodi M., Abbasi B., Daroudi M., Kazemi Oskuee R. (2019). Preparation of superparamagnetic iron oxide/doxorubicin loaded chitosan nanoparticles as a promising glioblastoma theranostic tool. J. Cell. Physiol..

[38] Ferrari M. (2008). Nanogeometry: Beyond drug delivery. Nat. Nanotechnol..

[39] Aksu Demirezen D., Yıldız Y.Ş., Yılmaz Ş., Demirezen Yılmaz D. (2019). Green synthesis and characterization of iron oxide nanoparticles using *Ficus carica* (common fig) dried fruit extract. J. Biosci. Bioeng..

[40] Singh K., Chopra D.S., Singh D., Singh N. (2020). Optimization and ecofriendly synthesis of iron oxide nanoparticles as potential antioxidant. Arab. J. Chem..

[41] Sarabaegi M., Roushani M. (2019). A nano-sized chitosan particle based electrochemical aptasensor for sensitive detection of: *P. aeruginosa*. Anal. Methods.

[42] Kumirska J., Czerwicka M., Kaczyński Z., Bychowska A., Brzozowski K., Thöming J., Stepnowski P. (2010). Application of spectroscopic methods for structural analysis of chitin and chitosan. Mar. Drugs.

[43] Mansur H.S., Sadahira C.M., Souza A.N., Mansur A.A.P. (2008). FTIR spectroscopy characterization of poly (vinyl alcohol) hydrogel with different hydrolysis degree and chemically crosslinked with glutaraldehyde. Mater. Sci. Eng. C.

[44] Silva V.A.J., Andrade P.L., Silva M.P.C., Bustamante A.D., De Los Santos Valladares L., Albino Aguiar J. (2013). Synthesis and characterization of Fe3O4 nanoparticles coated with fucan polysaccharides. J. Magn. Magn. Mater..

[45] Liu Y., Li P., Wang Y., Liu J., Wang Y., Zhang J., Wu M., Qiu J. (2017). A green and template recyclable approach to prepare Fe_3_O_4_/porous carbon from petroleum asphalt for lithium-ion batteries. J. Alloys Compd..

[46] Yen M., Yang J., Mau J. (2009). Physicochemical characterization of chitin and chitosan from crab shells. Carbohydr. Polym..

[47] Wang Y., Pitto-Barry A., Habtemariam A., Romero-Canelon I., Sadler P.J., Barry N.P.E. (2016). Nanoparticles of chitosan conjugated to organo-ruthenium complexes. Inorg. Chem. Front..

[48] Ma X.-D., Qian X.-F., Yin J., Xi H.-A., Zhu Z.-K. (2002). Preparation and Characterization of Polyvinyl Alcohol-Capped CdSe Nanoparticles at Room Temperature. J. Colloid Interface Sci..

[49] Sharma P., Mathur G., Goswami N., Sharma S.K., Dhakate S.R., Chand S., Mathur A. (2015). Evaluating the potential of chitosan/poly(vinyl alcohol) membranes as alternative carrier material for proliferation of Vero cells. E-Polymers.

[50] Li G.Y., Jiang Y.R., Huang K.L., Ding P., Chen J. (2008). Preparation and properties of magnetic Fe_3_O_4_-chitosan nanoparticles. J. Alloys Compd..

[51] Li X., Li Y., Zhang S., Ye Z. (2012). Preparation and characterization of new foam adsorbents of poly(vinyl alcohol)/chitosan composites and their removal for dye and heavy metal from aqueous solution. Chem. Eng. J..

[52] Miguel S.P., Moreira A.F., Correia I.J. (2019). Chitosan based-asymmetric membranes for wound healing: A review. Int. J. Biol. Macromol..

[53] Agarwal Y., Rajinikanth P.S., Ranjan S., Tiwari U., Balasubramnaiam J., Pandey P., Arya D.K., Anand S., Deepak P. (2021). Curcumin loaded polycaprolactone-/polyvinyl alcohol-silk fibroin based electrospun nanofibrous mat for rapid healing of diabetic wound: An in-vitro and in-vivo studies. Int. J. Biol. Macromol..

[54] Gauzit Amiel A., Palomino-Durand C., Maton M., Lopez M., Cazaux F., Chai F., Neut C., Foligné B., Martel B., Blanchemain N. (2020). Designed sponges based on chitosan and cyclodextrin polymer for a local release of ciprofloxacin in diabetic foot infections. Int. J. Pharm..

[55] Ibrahim F.Y., EL-Khateeb A.Y., Mohamed A.H. (2019). Rhus and Safflower Extracts as Potential Novel Food Antioxidant, Anticancer, and Antimicrobial Agents Using Nanotechnology. Foods.

[56] Guo S., DiPietro L.A. (2010). Critical review in oral biology & medicine: Factors affecting wound healing. J. Dent. Res..

[57] Hossain U., Das A.K., Ghosh S., Sil P.C. (2020). An overview on the role of bioactive α-glucosidase inhibitors in ameliorating diabetic complications. Food Chem. Toxicol..

[58] Mwakalukwa R., Amen Y., Nagata M., Shimizu K. (2020). Postprandial hyperglycemia lowering effect of the isolated compounds from olive mill wastes—An inhibitory activity and kinetics studies on α-glucosidase and α-amylase enzymes. ACS Omega.

[59] Bano F., Baber M., Ali A., Shah Z., Muhammad S.A. (2017). Biosynthesis, characterization, and biological activities of iron nanoparticles using *Sesamum indicum* seeds extract. Pharmacogn. Mag..

[60] Bowler P.G., Duerden B.I., Armstrong D.G. (2001). Wound microbiology and associated approaches to wound management. Clin. Microbiol. Rev..

[61] Arakha M., Pal S., Samantarrai D., Panigrahi T.K., Mallick B.C., Pramanik K., Mallick B., Jha S. (2015). Antimicrobial activity of iron oxide nanoparticle upon modulation of nanoparticle-bacteria interface. Sci. Rep..

[62] Chatterjee S., Bandyopadhyay A., Sarkar K. (2011). Effect of iron oxide and gold nanoparticles on bacterial growth leading towards biological application. J. Nanobiotechnol..

[63] Gabrielyan L., Hovhannisyan A., Gevorgyan V., Ananyan M., Trchounian A. (2019). Antibacterial effects of iron oxide (Fe_3_O_4_) nanoparticles: Distinguishing concentration-dependent effects with different bacterial cells growth and membrane-associated mechanisms. Appl. Microbiol. Biotechnol..

[64] Kong M., Chen X.G., Xing K., Park H.J. (2010). Antimicrobial properties of chitosan and mode of action: A state of the art review. Int. J. Food Microbiol..

[65] Olewnik-Kruszkowska E., Gierszewska M., Jakubowska E., Tarach I., Sedlarik V., Pummerova M. (2019). Antibacterial films based on PVA and PVA-chitosan modified with poly(hexamethylene guanidine). Polymers.

[66] Molecular Probes I. (2004). LIVE/DEAD ^®^ BacLight TM Bacterial Viability Kits. Prod. Inf..

[67] Gavard J., Hanini A., Schmitt A., Kacem K., Chau F., Ammar S., Gavard J. (2011). Evaluation of iron oxide nanoparticle biocompatibility. Int. J. Nanomed..

[68] Islam M.M., Shahruzzaman M., Biswas S., Nurus Sakib M., Rashid T.U. (2020). Chitosan based bioactive materials in tissue engineering applications—A review. Bioact. Mater..

[69] Bernal-Ballen A., Lopez-Garcia J.A., Ozaltin K. (2019). (PVA/chitosan/fucoidan)-ampicillin: A bioartificial polymeric material with combined properties in cell regeneration and potential antibacterial features. Polymers.

[70] Kamoun E.A., Chen X., Mohy Eldin M.S., Kenawy E.R.S. (2015). Crosslinked poly(vinyl alcohol) hydrogels for wound dressing applications: A review of remarkably blended polymers. Arab. J. Chem..

[71] Nour-Eldeen G., Abdel-Rasheed M., EL-Rafei A.M., Azmy O., El-Bassyouni G.T. (2020). Adipose tissue-derived mesenchymal stem cells and chitosan/poly (vinyl alcohol) nanofibrous scaffolds for cartilage tissue engineering. Cell Regen..

[72] Kegere J., Ouf A., Siam R., Mamdouh W. (2019). Fabrication of Poly(vinyl alcohol)/Chitosan/Bidens pilosa Composite Electrospun Nanofibers with Enhanced Antibacterial Activities. ACS Omega.

[73] Ali L.M.A., Shaker S.A., Pinol R., Millan A., Hanafy M.Y., Helmy M.H., Kamel M.A., Mahmoud S.A. (2020). Effect of superparamagnetic iron oxide nanoparticles on glucose homeostasis on type 2 diabetes experimental model. Life Sci..

[74] Sharifi S., Daghighi S., Motazacker M.M., Badlou B., Sanjabi B., Akbarkhanzadeh A., Rowshani A.T., Laurent S., Peppelenbosch M.P., Rezaee F. (2013). Superparamagnetic iron oxide nanoparticles alter expression of obesity and T2D-associated risk genes in human adipocytes. Sci. Rep..

[75] Jeong S., Min Cho J., Kwon Y.I., Kim S.C., Yeob Shin D., Ho Lee J. (2019). Chitosan oligosaccharide (GO2KA1) improves postprandial glycemic response in subjects with impaired glucose tolerance and impaired fasting glucose and in healthy subjects: A crossover, randomized controlled trial. Nutr. Diabetes.

[76] Kim J.A., Lee N., Kim B.H., Rhee W.J., Yoon S., Hyeon T., Park T.H. (2011). Enhancement of neurite outgrowth in PC12 cells by iron oxide nanoparticles. Biomaterials.

[77] Ziv-Polat O., Topaz M., Brosh T., Margel S. (2010). Enhancement of incisional wound healing by thrombin conjugated iron oxide nanoparticles. Biomaterials.

[78] Jin R., Liu L., Zhu W., Li D., Yang L., Duan J., Cai Z., Nie Y., Zhang Y., Gong Q. (2019). Iron oxide nanoparticles promote macrophage autophagy and inflammatory response through activation of toll-like Receptor-4 signaling. Biomaterials.

[79] Akbar M.U., Zia K.M., Akash M.S.H., Nazir A., Zuber M., Ibrahim M. (2018). In-vivo anti-diabetic and wound healing potential of chitosan/alginate/maltodextrin/pluronic-based mixed polymeric micelles: Curcumin therapeutic potential. Int. J. Biol. Macromol..

[80] Sathiyaseelan A., Saravanakumar K., Mariadoss A.V.A., Ramachandran C., Hu X., Oh D.H., Wang M.H. (2020). Chitosan-tea tree oil nanoemulsion and calcium chloride tailored edible coating increase the shelf life of fresh cut red bell pepper. Prog. Org. Coat..

[81] Saravanakumar K., Mariadoss A.V.A., Sathiyaseelan A., Wang M.H. (2020). Synthesis and characterization of nano-chitosan capped gold nanoparticles with multifunctional bioactive properties. Int. J. Biol. Macromol..

[82] Sathiyaseelan A., Saravanakumar K., Mariadoss A.V.A., Wang M.H. (2020). Biocompatible fungal chitosan encapsulated phytogenic silver nanoparticles enhanced antidiabetic, antioxidant and antibacterial activity. Int. J. Biol. Macromol..

[83] Wiegand I., Hilpert K., Hancock R.E.W. (2008). Agar and broth dilution methods to determine the minimal inhibitory concentration (MIC) of antimicrobial substances. Nat. Protoc..

[84] Saravanakumar K., Park S., Sathiyaseelan A., Kim K.-N., Cho S.-H., Mariadoss A.V.A., Wang M.-H. (2021). Metabolite Profiling of Methanolic Extract of Gardenia jaminoides by LC-MS/MS and GC-MS and Its Anti-Diabetic, and Anti-Oxidant Activities. Pharmaceuticals.

[85] Mariadoss A.V.A., Park S., Saravanakumar K., Sathiyaseelan A., Wang M.-H. (2021). Ethyl Acetate Fraction of Helianthus tuberosus L. Induces Anti-Diabetic, and Wound-Healing Activities in Insulin-Resistant Human Liver Cancer and Mouse Fibroblast Cells. Antioxidants.

[86] Varankar S.S., Bapat S.A. (2018). Migratory Metrics of Wound Healing: A Quantification Approach for in vitro Scratch Assays. Front. Oncol..

[87] Pongrac I.M., Radmilović M.D., Ahmed L.B., Mlinarić H., Regul J., Škokić S., Babič M., Horák D., Hoehn M., Gajović S. (2019). D-mannose-Coating of Maghemite Nanoparticles Improved Labeling of Neural Stem Cells and Allowed Their Visualization by ex vivo MRI after Transplantation in the Mouse Brain. Cell Transplant..

