# Supplementary material for: Antimicrobial and Wound Healing Properties of FeO Fabricated Chitosan/PVA Nanocomposite Sponge
Source: Antibiotics (Basel). 2021 May 3;10(5):524. doi: 10.3390/antibiotics10050524 (PMC8147619; doi:10.3390/antibiotics10050524)
Supplement: Supplementary file 1 [file antibiotics-10-00524-s001.zip › antibiotics-1199839-SI.pdf]

antibiotics

MDPI

Article

Antimicrobial and Wound Healing Properties of FeO Fabricated Chitosan/PVA Nanocomposite Sponge

Anbazhagan Sathiyaseelan, Kandasamy Saravanakumar, Arokia Vijay Anand Mariadoss and Myeong-Hyeon Wang *

Department of Bio-Health Convergence, Kangwon National University, Chuncheon 200-701, Korea; sathiyaseelan.bio@gmail.com (A.S.); saravana732@gmail.com (K.S.); mavijaibt@gmail.com (A.V.A.M.)
* Correspondence: mhwang@kangwon.ac.kr

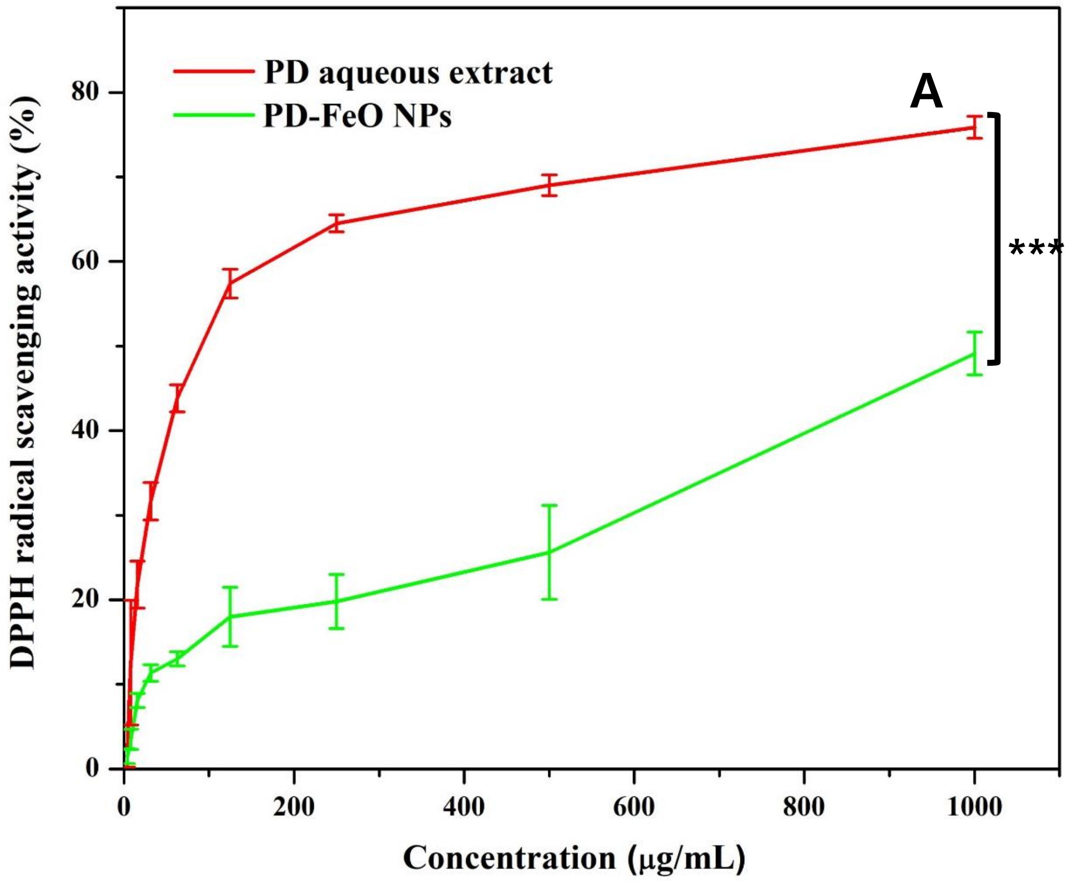

Figure S1. DPPH radical scavenging activity. Aqueous extract of *Pinus densiflora* (PD aqueous extract) and PD aqueous extract mediated iron oxide nanoparticles (PD-FeO NPs). *** $p<0.001$.

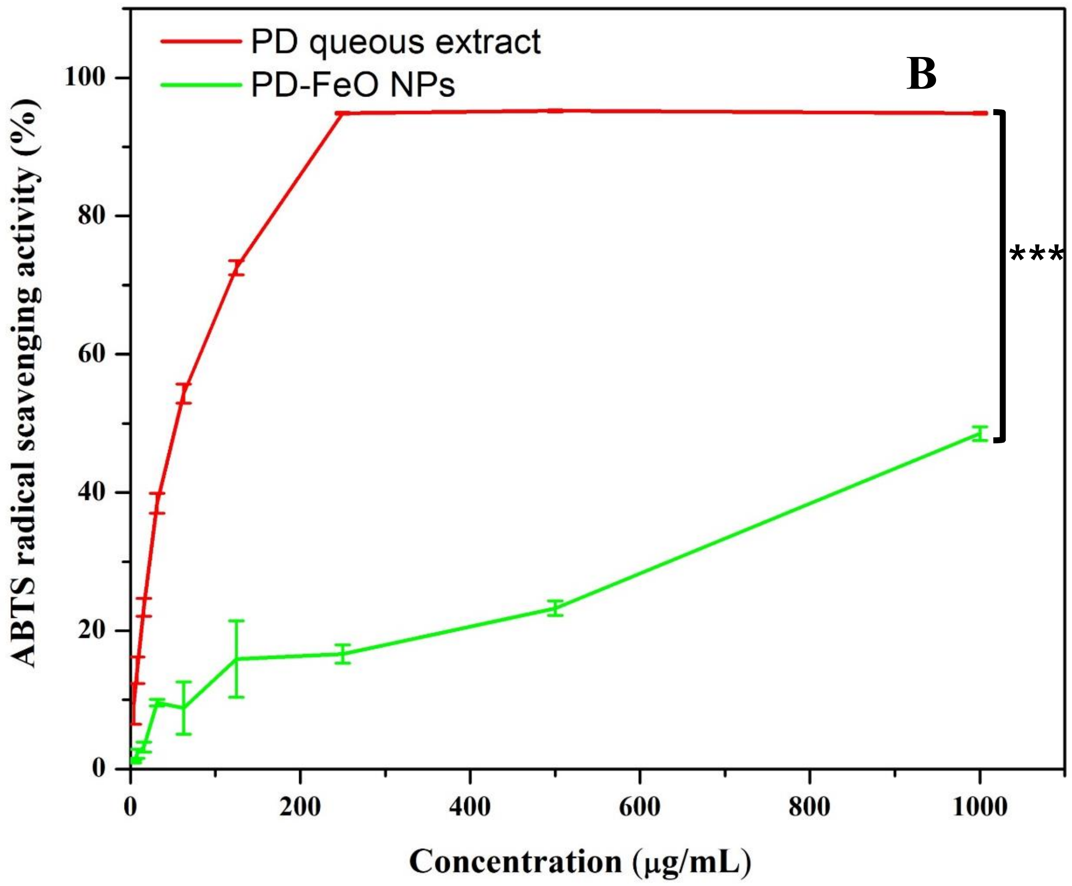

Figure S2. ABTS radical scavenging activity. Aqueous extract of *Pinus densiflora* (PD aqueous extract) and PD aqueous extract mediated iron oxide nanoparticles (PD-FeO NPs). *** $p<0.001$.

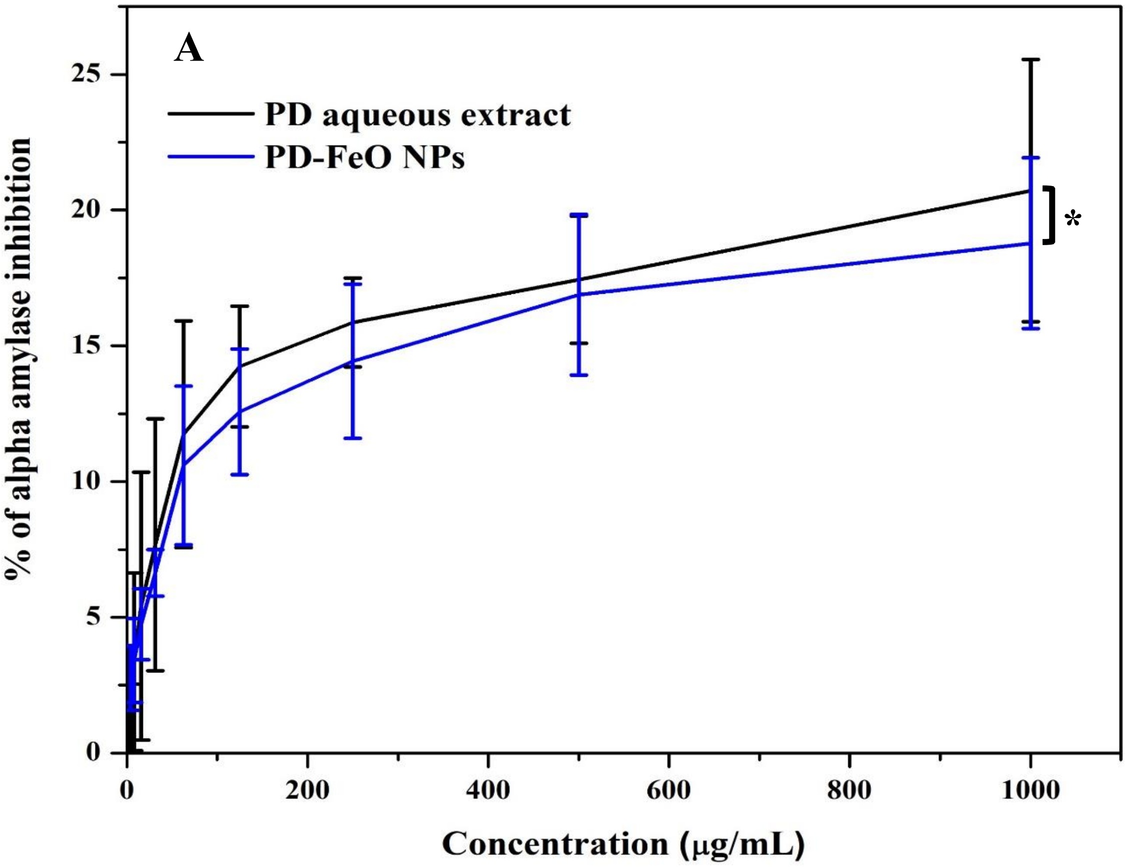

Figure S3. Alpha-glucosidase enzyme inhibitory activity. Aqueous extract of *Pinus densiflora* (PD aqueous extract) and PD aqueous extract mediated iron oxide nanoparticles (PD-FeO NPs). * $p<0.05$ significant.

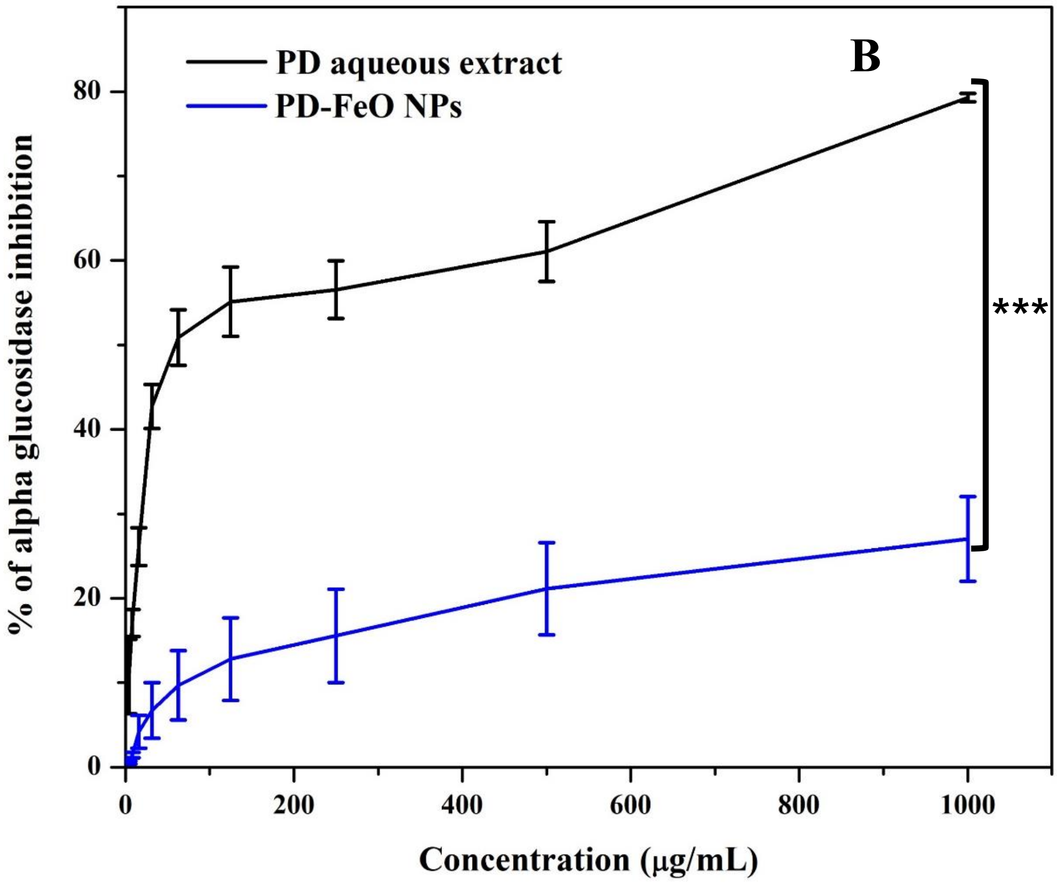

Figure S4. Alpha-amylase enzyme inhibitory activity. Aqueous extract of *Pinus densiflora* (PD aqueous extract) and PD aqueous extract mediated iron oxide nanoparticles (PD-FeO NPs). *** $p<0.001$.

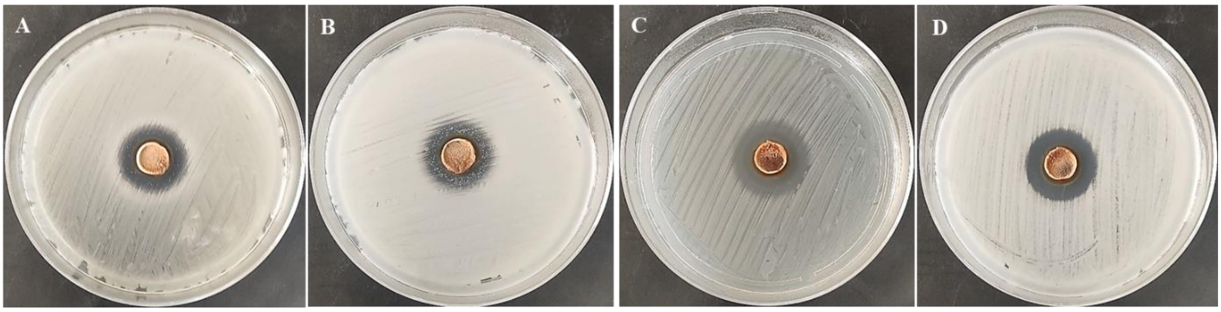

Figure S5. Antibacterial activity of CS/PVA-PD-FeO NPs sponges. *Bacillus cereus – A; Staphylococcus aureus - B; Escherichia coli – C; Salmonella enterica – D.*

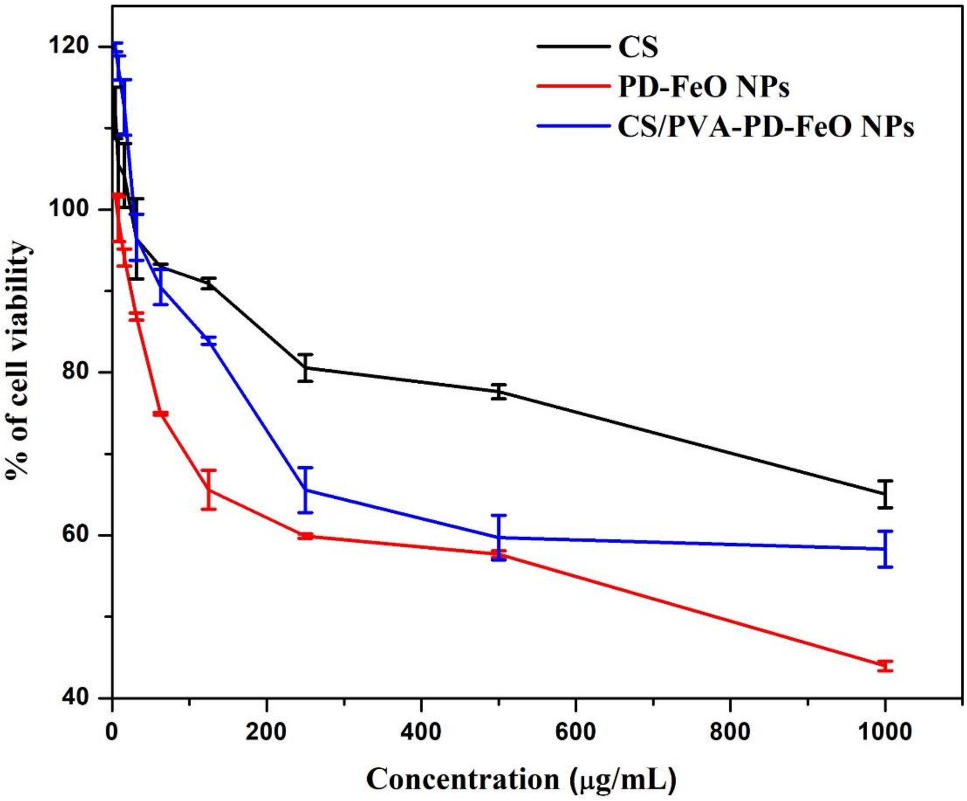

Figure S6. Cell viability. HEK293 cells treated with CS, PD-FeO NPS, and CS/PVA-PD-FeO NPs (0.01%).
